# Seasonal decline in leaf photosynthesis in perennial switchgrass explained by sink limitations and water deficit

**DOI:** 10.3389/fpls.2022.1023571

**Published:** 2023-01-04

**Authors:** Mauricio Tejera-Nieves, Michael Abraha, Jiquan Chen, Stephen K. Hamilton, G. Philip Robertson, Berkley James Walker

**Affiliations:** ^1^ MSU-DOE Plant Research Laboratory, Michigan State University, East Lansing, MI, United States; ^2^ Great Lakes Bioenergy Research Center, Michigan State University, East Lansing, MI, United States; ^3^ W. K. Kellogg Biological Station, Michigan State University, Hickory Corners, MI, United States; ^4^ Center for Global Change and Earth Observations, Michigan State University, East Lansing, MI, United States; ^5^ Department of Geography, Environment, and Spatial Sciences, Michigan State University, East Lansing, MI, United States; ^6^ Department of Integrative Biology, Michigan State University, East Lansing, MI, United States; ^7^ Department of Plant, Soil, and Microbial Sciences, Michigan State University, East Lansing, MI, United States; ^8^ Department of Plant Biology, Michigan State University, East Lansing, MI, United States

**Keywords:** photosyhthesis, storage carbohydrates, sink limitation, perennial grass, drought, circadian, source- sink- relationships, C4 photosynthesis

## Abstract

Leaf photosynthesis of perennial grasses usually decreases markedly from early to late summer, even when the canopy remains green and environmental conditions are favorable for photosynthesis. Understanding the physiological basis of this photosynthetic decline reveals the potential for yield improvement. We tested the association of seasonal photosynthetic decline in switchgrass (*Panicum virgatum* L.) with water availability by comparing plants experiencing ambient rainfall with plants in a rainfall exclusion experiment in Michigan, USA. For switchgrass exposed to ambient rainfall, daily net CO_2_ assimilation ( $A_{net}^{'}$ ) declined from 0.9 mol CO_2_ m^-2^ day^-1^ in early summer to 0.43 mol CO_2_ m^-2^ day^-1^ in late summer (53% reduction; P<0.0001). Under rainfall exclusion shelters, soil water content was 73% lower and $A_{net}^{'}$ was 12% and 26% lower in July and September, respectively, compared to those of the rainfed plants. Despite these differences, the seasonal photosynthetic decline was similar in the season-long rainfall exclusion compared to the rainfed plants; $A_{net}^{'}$ in switchgrass under the shelters declined from 0.85 mol CO_2_ m^-2^ day^-1^ in early summer to 0.39 mol CO_2_ m^-2^ day^-1^ (54% reduction; P<0.0001) in late summer. These results suggest that while water deficit limited $A_{net}^{'}$ late in the season, abundant late-season rainfalls were not enough to restore $A_{net}^{'}$ in the rainfed plants to early-summer values suggesting water deficit was not the sole driver of the decline. Alongside change in photosynthesis, starch in the rhizomes increased 4-fold (P<0.0001) and stabilized when leaf photosynthesis reached constant low values. Additionally, water limitation under shelters had no negative effects on the timing of rhizome starch accumulation, and rhizome starch content increased ~ 6-fold. These results showed that rhizomes also affect leaf photosynthesis during the growing season. Towards the end of the growing season, when vegetative growth is completed and rhizome reserves are filled, diminishing rhizome sink activity likely explained the observed photosynthetic declines in plants under both ambient and reduced water availability.

## Introduction

Leaf photosynthesis of perennial grasses follows a clear seasonal dynamic – peaking with vegetative growth in summer and declining towards late summer several weeks before the end of the growing season while leaves are still green. This dynamic occurs in many genera of grasses including the bioenergy crops *Panicum*, *Miscanthus*, and *Saccharum* spp., and even in some evergreen and deciduous trees (Eggemeyer et al., 2006; Kosugi and Matsuo, 2006; Inman-Bamber et al., 2011; De Souza et al., 2013; Boersma et al., 2015; Gao et al., 2015; Yan et al., 2015; De Souza et al., 2018; Endres et al., 2019; Rusinowski et al., 2019; Stavridou et al., 2020). This seasonal decline in photosynthesis could be partially adaptative as it is associated with the remobilization of nutrients from leaves to belowground perennating organs, which allows the plant to recycle nutrients that otherwise would be lost to leaf drop (Yang et al., 2009; Yang et al., 2016; Yang and Udvardi, 2018; Massey et al., 2020). Beyond this putative adaptive role in nutrient retranslocation, additional physiological and environmental drivers of this decrease in photosynthesis are unknown but could have large effects on end-of-season biomass and long-term yield dynamics.

Perennial grasses notably increase in size during the growing season, with larger plants being progressively more susceptible to water limitation as a larger transpiring leaf area increases total water demand and increasingly depletes available soil water (Liu et al., 2016; Mocko and Jones, 2021; Wang et al., 2021). Therefore, the observed photosynthetic decline could be a consequence of water limitation as plants become larger and soil water becomes less available later in the growing season. Alternatively, the larger size could also lead to a nitrogen (N) limitation (Leroy et al., 2022), however, this decline was not affected by N availability across a wide range of N fertilization rates (Tejera et al., 2022).

Among perennial grasses, switchgrass (*Panicum virgatum* L.) is a leading candidate for cellulosic bioenergy feedstock in the United States (Parrish and Fike, 2005). Evidence increasingly supports switchgrass as a drought tolerant species, based on its leaf photosynthesis and plant growth resiliency in the face of water limitation (Barney et al., 2009; Lovell et al., 2016; Taylor et al., 2016; Hawkes and Kiniry, 2018; Chen et al., 2020). This suggests that water limitation may not drive the photosynthesis decline observed late in the growing season. However, previous water limitation studies mainly focused on the initial photosynthetic response to drought (Lovell et al., 2016; Taylor et al., 2016; Chen et al., 2020), and the response through the entire growing season is not as well resolved. Measuring drought effects on photosynthesis over the growing season is costly as it requires large rainfall-exclusion shelters in field experiments. Additionally, these experiments usually rely on natural precipitation events as control treatments, reducing the power of the experiment to detect water-deficit effects during dry periods. Despite these drawbacks, rainfall-exclusion experiments can be more realistic than controlled irrigation studies performed in greenhouses because field experiments include other environmental factors (Lovell et al., 2016).

Switchgrass leaf photosynthesis response to water limitation is mainly studied at peak light availability (Lovell et al., 2016; Taylor et al., 2016; Chen et al., 2020). While measurements at peak solar irradiance are useful to characterize photosynthesis when heat stress is maximal, they do not capture diurnal changes in light and water availability. These point measurements thus fail to incorporate the midday or early afternoon periods of photosynthesis depression observed in other perennials (Gao et al., 2015; Bucci et al., 2019), and overlook changes in the source-sink dynamic during the day.

The late-season photosynthesis decline could also be driven by co-occurring physiological changes. Specifically, physiological signals like carbohydrate buildup and sink strength are known to limit development and leaf photosynthesis in certain perennial grasses (McCormick et al., 2009; Van Heerden et al., 2010; De Souza et al., 2018; Ruiz-Vera et al., 2021; Tejera et al., 2021; Tejera et al., 2022) . For example, when the photoassimilate source-sink balance was perturbed by shading some leaves of sugarcane plants, photosynthesis in the unshaded leaves increased by 32%, indicating source-sink coordination at the level of the entire plant to match supply (source) with demand (sinks) (McCormick et al., 2006). Similarly, when sink strength was reduced by cold-girdling leaves or by placing leaves in sucrose solution, photosynthesis decreased by 30 – 45%, probably inhibited by foliar sucrose and hexose concentrations that increased 2 – 3 fold (McCormick et al., 2008). These studies reveal the short-term effects of source/sink relationships on photosynthetic rates in perennial systems but fail to explain seasonal dynamics and environmental limitations. The late-season decrease in photosynthesis could be caused by sink limitations in carbohydrate storage organs (Van Heerden et al., 2010; De Souza et al., 2018). As the season progresses, major sink organs and processes (i.e., growth, storage refill, reproduction) cease activity and limit their carbohydrate consumption, which would then lead to a lower demand for photosynthate and ultimately reducing leaf photosynthesis (Tejera et al., 2022).

Water limitation also imposes restrictions on carbon sink organs and processes that may interact with carbohydrate buildup and photosynthesis limitations (Lemoine et al., 2013; Rodrigues et al., 2019). On the one hand, water limitation causes a decrease in photosynthesis which in turn may lead to depletion of carbohydrate in belowground storage organs (Barney et al., 2009; Lovell et al., 2016; Taylor et al., 2016; Hawkes and Kiniry, 2018; Ivanov et al., 2019; Chen et al., 2020). After water stress is ameliorated, rhizomes would then resume carbohydrate accumulation, allowing photosynthesis to persist for longer in the season. On the other hand, water limitation also reduces above- and below-ground growth (Barney et al., 2009; Mann et al., 2013; Hui et al., 2018), which could cause additional sink limitations. In this case, the carbohydrate buildup in the rhizomes would diminish earlier in the season, with corresponding declines in switchgrass photosynthesis.

To resolve these interacting and potentially conflicting effects of source-sink relations and water deficit on the seasonal changes of photosynthesis, we studied the seasonal dynamics of photosynthesis in switchgrass, representing a fast-growing perennial grass, under rainfed and experimentally induced water limitation treatments. Specifically, we asked: 1) Does switchgrass photosynthesis correlate with rhizome sink strength on a seasonal basis under conditions of ambient rainfall and soil water availability? and 2) Does experimentally imposed season-long water limitation affect seasonal patterns of photosynthesis and sink strength, and particularly does it accelerate the onset of the observed late-season photosynthesis decline? To address these questions, we measured switchgrass source activity (i.e., diurnal course of photosynthesis) and sink strengths of carbohydrates in source (leaves) and sink (rhizome) organs in mature switchgrass stands across an entire growing season. The expected seasonal decrease in leaf photosynthesis, found in both plot and field-scale switchgrass stands, co-occurred with peak carbohydrate concentrations in both leaves and rhizomes. Water limitation reduced switchgrass leaf photosynthesis in July and September but, abundant late-season rainfalls were not enough to restore $A_{net}^{'}$ in the rainfed plants to early-summer values. Additionally, water limitations had no effects on rhizome starch dynamics and starch in the rhizomes increased ~ 6-fold. These results suggest that while water deficit limited $A_{net}^{'}$ in the late season, late-season precipitations did not restore $A_{net}^{'}$ to early-summer values suggesting that other limitations were also in place. Towards the end of the growing season, when vegetative growth is completed and rhizome reserves are filled, the insufficient sink activity presents a strong limitation, leading to the observed photosynthetic decline.

## Materials and methods

### Experimental design

Water limitation was imposed by placing rainfall exclusion shelters over switchgrass plots that are part of the Biofuel Cropping System Experiment (BCSE) of the Great Lakes Bioenergy Research Center (GLBRC), located at the Kellogg Biological Station (KBS) Long-term Ecological Research site in Hickory Corners, Michigan, USA (42.394290 N, -85.374126 W). For our experiment, we used four replicate switchgrass plots. Switchgrass (cv. Cave-in-rock) was planted in June 2008 at a seeding rate of 7.71 kg ha^-1^ and row spacing of 0.2 m and received 56 kg N ha^-1^ yr^-1^ after its initial establishment year (Sanford et al., 2016). The switchgrass was re-seeded in 2009 due to intense storms in mid-summer 2008 that redistributed un-germinated seeds. Each plot was 30 x 40 m and had a rainfall exclusion shelter that measured 4.2 x 5.5 m and 2.6 m tall located at least 1 m inside the plot. Corrugated roofing panels (Greca Lexan; Amerilux, De Pere, WI, USA) allowed ~90% light transmittance (385-700 nm). In 2020, the year of sampling, rainfall exclusion shelters were in place from May 27 – September 10. In 2019 rainfall exclusion shelters had been in place over the same footprints from May 24 – June 22 and September 9 – October 11, and in 2018 from June 13 – October 24. Soil volumetric water content (VWC) sensors (CS655; Campbell Scientific Inc. CSI, Logan, UT, USA) were installed in two plots horizontally at 0.10 m and 0.25 m depth under the rainfall exclusion shelter, and in the open field within 4m radius of the shelter.

### Data and sample collection

We sampled the experiment five times during the 2020 growing season (19 June, 1 July, 28 July 6 August, and 3 September) to cover the entire switchgrass growth cycle. For the first two sampling dates switchgrass stands were in vegetative stages, for the third and fourth dates the stands were in reproductive stages, and for the last date the stands were >75% senesced. On each sampling date, we sampled plants outside the rainfall exclusion shelter (Rainfed treatment) and under the shelters (Rainfall exclusion shelter treatment) from each plot. Rainfall exclusion shelter samples were taken from a sampling area of 1 m^2^. Rainfed samples were collected from two sampling areas separated 4 m from each other. A 1-m^2^ end-of-season biomass sample was collected on November 3, 2020, from the center portion of each shelter, as well as from the rainfed sampling locations. Samples were dried at 60°C until constant moisture and weighed to estimate the dry biomass.

On each sampling date, we conducted a diurnal sampling of photosynthetic rate, leaf water potential (LWP), and carbohydrate content in source (leaves) and sink (rhizome) organs. The first and last timepoints of the diurnal samplings were collected before sunrise and after sunset. Three timepoints were collected during the day: at mid-morning (9:00 – 10:00 h), noon (13:00 – 14:00 h) and mid-afternoon (17:00 – 18:00 h). We sampled rhizomes before sunrise, around solar noon, and after sunset. Leaf net CO_2_ assimilation rate ( *A*_*net*_ ) and stomatal conductance to water (g_sw_) were measured in the middle portion of the youngest fully expanded leaf using an open gas exchange system (Li-6800; LI-COR Biosciences, Lincoln, NE, USA) equipped with an integrated modulated chlorophyll fluorometer and a light source. Air temperature, photosynthetic photon flux density (PPDF), and relative humidity (RH) inside the leaf chamber were set to mimic ambient conditions at each sampling time The Li-6800 controls the leaf environment using internal LED lighting, Peltier heating/cooling units and controlling incoming CO_2_ and H_2_O content using a series of chemical scrubbing columns. The CO_2_ concentration was maintained at 400 μmol mol^–1^. The diurnal sum of *A*_*net*_ ( $A_{net}^{'}$ ) was calculated using the area under the curve (AUC), with sunrise and sunset as the limits of integration. Non-photochemical quenching and dark-adapted Photosystem II (PSII) maximum quantum yield (F_v_/F_m_) values were measured using chlorophyll fluorescence from dark-adapted leaves at the pre-dawn sampling.

PSII quantum efficiency ( *ϕPSII* ) was calculated as:


$$\phi PSII=\frac{F_{m}^{'}-\;F_{s}}{F_{m}^{'}}$$

where *F*_*s*_ is the steady-state fluorescence, and $F_{m}^{'}$ is the maximum fluorescence after a saturating light flash.

At each sampling in each plot, we evaluated two leaf samples for a total of 16 samples per sampling, 80 per sampling date, and 400 for the entire experiment. The same leaf was first measured with the Li-6800, then placed in a pressure chamber (model 1505D; PMS Instruments, Albany, OR, USA) equipped with a grass compression gland to measure its leaf water potential (LWP), and then flash-frozen in liquid nitrogen (N) within 5 min of harvest. A small rhizome sample (~3 g) was harvested from the same stem that bore the leaf and immediately frozen in liquid N. In all samplings, rhizomes were clearly distinguished from stems and newly formed tillers (**Figure S1**). All samples were kept in liquid N until the next day and then stored at -80°C until further processing.

### Sample processing

Leaf samples were ground to a fine powder with a mortar and pestle. Rhizome samples were ground with a spice mixer (Cuisinart; SG-10). All samples stayed frozen during grinding and were then freeze-dried for at least 48 hours in a lyophilizer. Diurnal concentrations of starch, sucrose and free glucose were measured in leaves ( [*starch*]_*leaf*_, [*suc*]_*leaf*_ , [*glu*]_*leaf*_ , respectively) and rhizomes ( [*starch*]_*rhi*_, [*suc*]_*rhi*_ , [*glu*]_*rhi*_ ) for the five sampling dates. Diurnal accumulation rates of these carbohydrates were estimated as the slope of the linear regression of predawn and daylight values over time. All assays were performed at the Biomass Analytics Facility at Michigan State University following procedures described in Santoro et al. (2010) and Sekhon et al. (2016). In brief, glucose content was assayed using the glucose oxidase/peroxidase (GOPOD) method (K-GLUC, Megazyme, Ireland). To determine sucrose and starch concentrations, samples were first treated with a combination of alkaline buffer and high heat to degrade all pre-existing free glucose. Then samples were treated with invertase (Sigma-Aldrich, St. Louis, MO), or amyloglucosidase (K-TSTA, Megazyme, Ireland) and 5 μL α-amylase (K-TSTA, Megazyme) for sucrose and starch extraction, respectively. The rest of the processing was identical to that used for glucose.

### Canopy-level gross CO_2_ assimilation

Gross primary production (GPP), defined here as canopy-level gross CO_2_ assimilation (canopy *A*_*gross*_ ), was estimated from eddy covariance tower net ecosystem CO_2_ exchange (NEE) observations in an 18-ha switchgrass stand of the same age with similar soil type and management at the Lux Arbor reserve (42.476100N, -85.446945W), 11 km from the experimental site (Abraha et al., 2018). NEE was measured at 10 Hz using an LI-7500 open-path infrared gas analyzer (LI-COR Biosciences) and a CSAT3 three-dimensional sonic anemometer (Campbell Scientific Inc.). The raw data were processed to compute 30-min NEEs. The 30-min NEEs were gap-filled and partitioned into ecosystem respiration (R_eco_) and GPP using the nighttime partitioning method in REddyProc package (Reichstein et al., 2005; Wutzler et al., 2018). The method assumes NEE is equal to R_eco_, and GPP is zero during the night. R_eco_ is then estimated – for both day and nighttime – from air temperature and nighttime R_eco_ relationship, and GPP is computed as a residual of the estimated R_eco_ and NEE (Abraha et al., 2018). Air temperature, incident solar radiation and vapor pressure deficit were also measured at the site.

### Data analysis

We used R software (R Core Team, 2017) for all analyses and plots. All models had the same random structure. We used lmer() in the lme4 package (Bates et al., 2015) to fit the mixed models, Anova() in the car package (Fox and Weisberg, 2011) for analysis of deviance using type II Wald chi-square test, and emmeans() in the emmeans package (Lenth et al., 2018) for mean and slope comparisons.

## Results

### Leaf photosynthesis decreased by ~50% in the latter half of the growing season.

**Figure 1:**
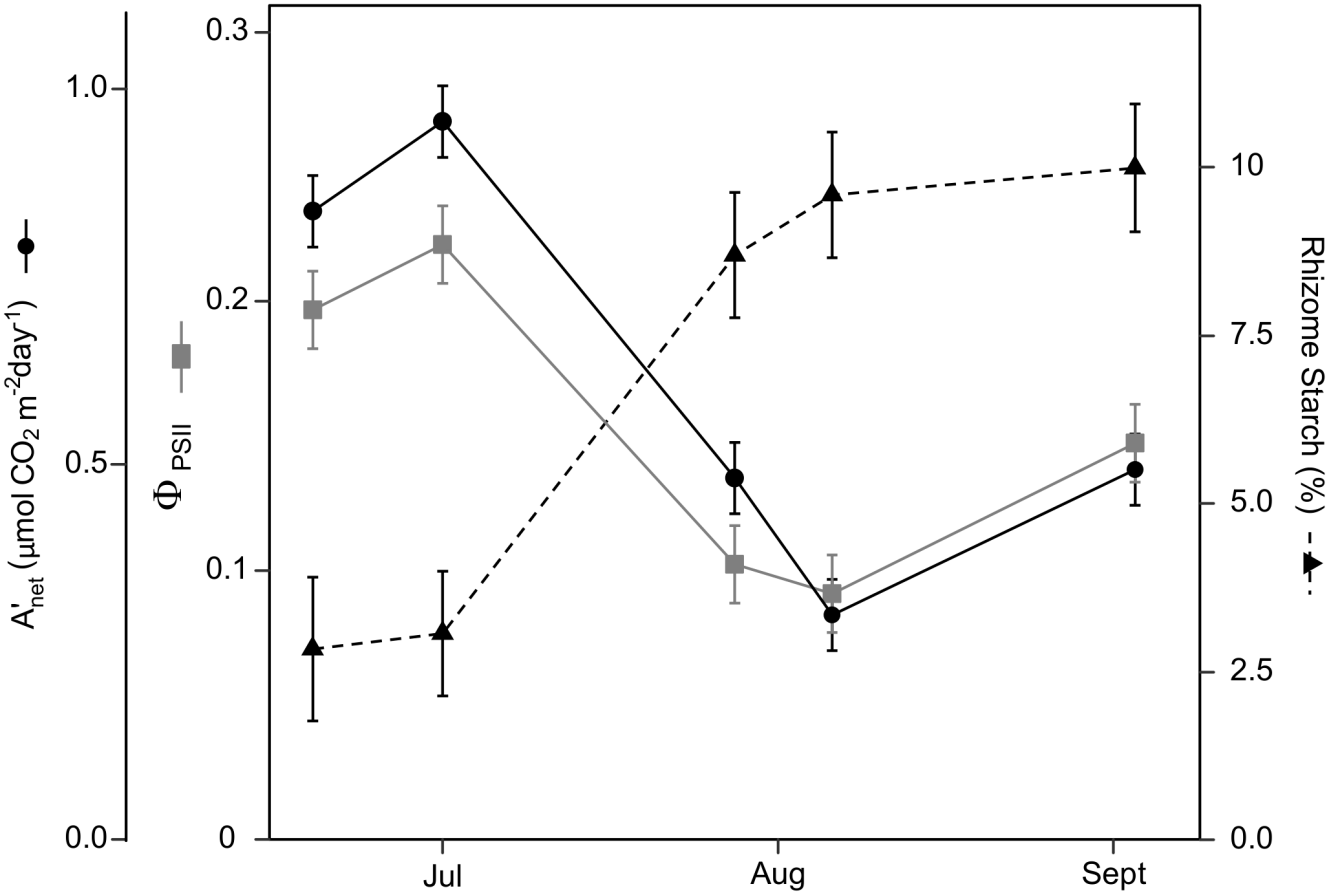
Switchgrass daily net CO_2_ assimilation ( $A_{net}^{'}$ ), efficiency of photosystem II ( _*PSII*_ ), and rhizome starch content during 2020. As the season progressed $A_{net}^{'}$ and _*PSII*_  decreased by ~50%, while rhizome starch increased 4-fold. During the first two sampling dates, switchgrass was in vegetative stages, the next two was in reproductive stages and in the last measurement switchgrass was 70% senesced. Error bars represent plus/minus one standard error of the mean (n = 4).

**Figure 2:**
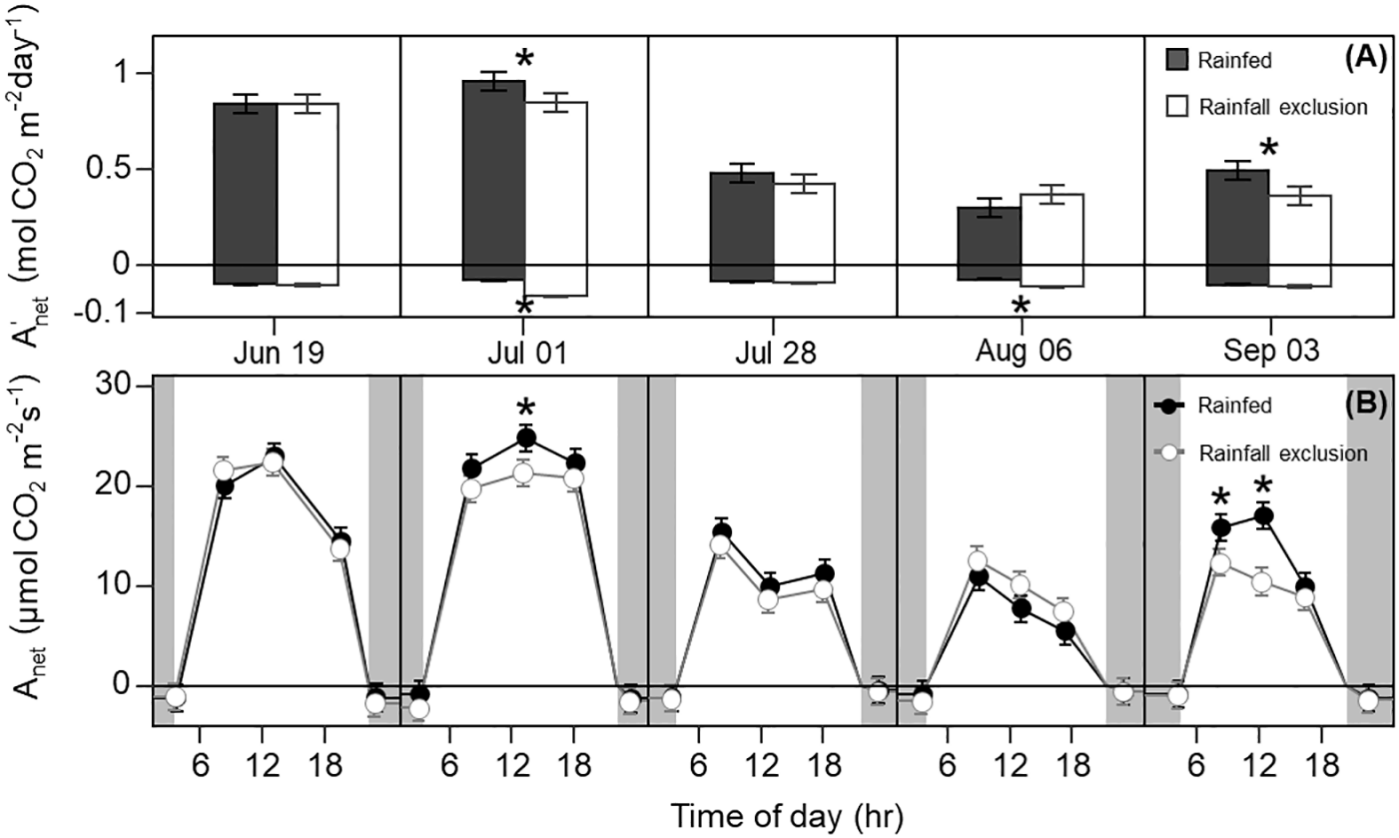
Switchgrass daily net CO_2_ assimilation ( $A_{net}^{'}$ ; A) integrated over each sampling date, and net CO_2_ assimilation rate ( $A_{\mathrm{net}}$ ; B) at each timepoint during the day, for plants grown under (white fill, grey line) and outside (black fill, black line) rainfall exclusion shelters. Negative values indicate dark respiration during the night period **(A)**, and dark respiration at predawn and post-dusk timepoints **(B)**. Asterisks indicate a significant difference between treatments (P< 0.05). Data are mean ± S.E. (n = 4).

**Figure 3:**
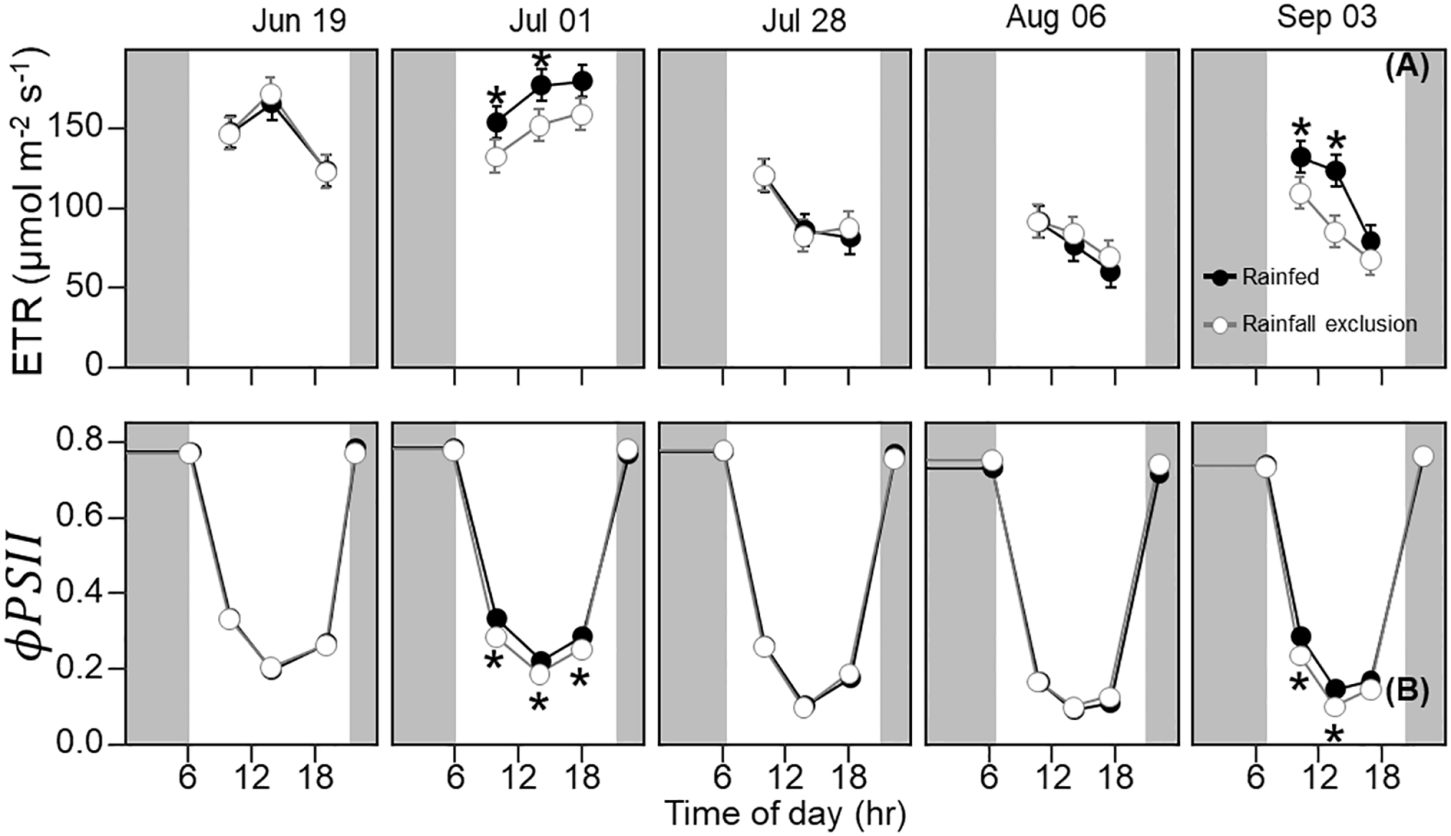
Switchgrass electron transport rate ( *ETR* ; **(A)** and photosystem II quantum efficiency ( *ϕPSII* ; **(B)** at each timepoint during the day, over the course of the growing season, for plants grown under (white fill, grey line) and outside (black fill, black line) rainfall exclusion shelters. Asterisks indicate a significant difference between treatments (P< 0.05). Data are mean ± S.E. (n = 4).

Leaf photosynthesis decreased by ~50% in the latter half of the growing season. Under ambient rainfall, switchgrass photosynthesis during the growing season markedly decreased by late July, prior to visible canopy senescence. Specifically, $A_{net}^{'}$ decreased from 0.84 – 0.96 mol CO_2_ m^-2^ day^-1^ in early summer (19 June and 1 July) to 0.30 – 0.50 mol CO_2_ m^-2^ day^-1^ in late summer (28 July, 6 August and 3 September; **Figures 1**, **2**). This ~53% reduction in $A_{net}^{'}$ from early to late summer (P< 0.001) was contributed from the consistently low *A*_*net*_ values throughout the day (**Figure 2**; P< 0.001). Changes in *A*_*net*_ or $A_{net}^{'}$ were not explained by increases in leaf respiration because nocturnal respiration rates changed little during the season, with no significant differences found between early and late summer (**Figure 2A**; P = 0.90). Corresponding with changes in *A*_*net*_ , *ϕPSII* and the electron transport rate (ETR) had similar seasonal pattern, with mid-morning, noon and mid-afternoon *ϕPSII* at 29 – 45% lower in late-summer (**Figure 3**; P< 0.001).

### Canopy-level CO_2_ gross assimilation decreased by ~34% from June to September

**Figure 4:**
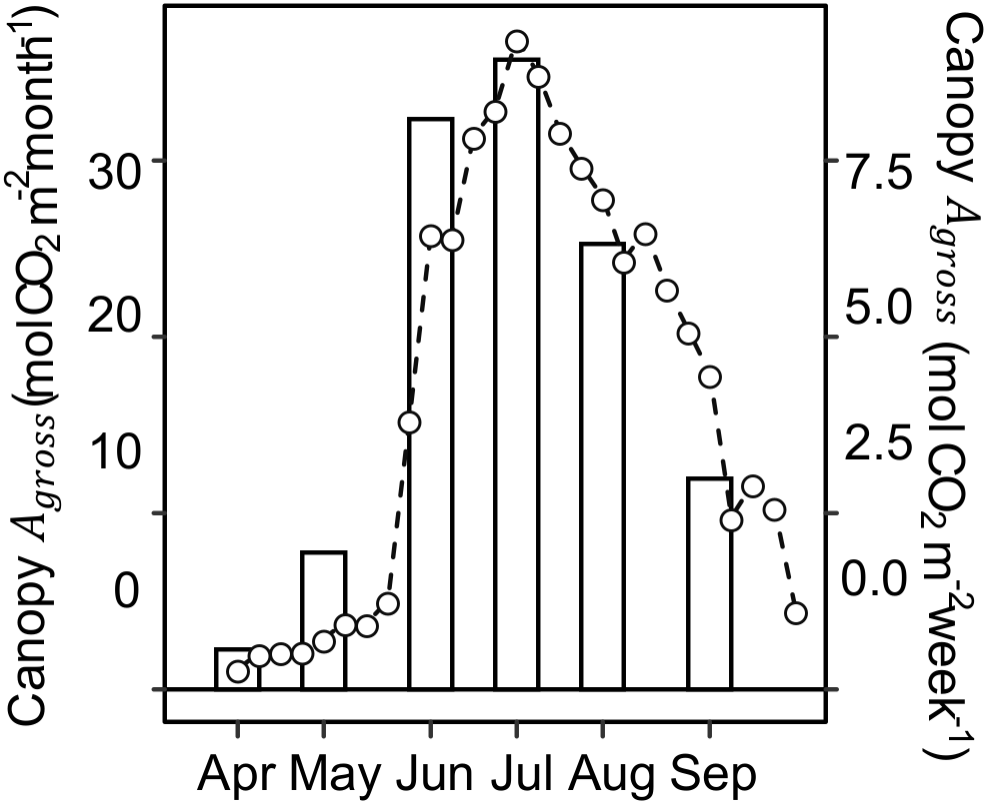
Monthly (bars) and weekly (lines and open circles) canopy gross CO_2_ assimilation (Canopy *A*_*gross*_ ) during the 2020 growing season. Canopy *A*_*gross*_  was computed from net ecosystem CO_2_ exchange (NEE) observations using eddy covariance method conducted at a switchgrass field of the same age as the experimental site but located 11 km away.

Canopy photosynthesis under ambient rainfall as measured by eddy covariance at the Lux Arbor switchgrass stand peaked in July and steadily decreased during the second half of the growing season (**Figure 4**). Specifically, Canopy *A*_*gross*_ decreased by ~34%, from ~1.24 mol CO_2_ m^-2^ day^-1^ in early-summer (June 19 – July 1) to ~0.82 mol CO_2_ m^-2^ day^-1^ in late-summer (July 28 – September 3; **Figure S2**). Canopy *A*_*gross*_ and leaf *A*_*net*_ were highly correlated both as individual measurements and daily accumulation (P<0.0001; **Figure S3**), indicating that the observed decline at the leaf level in the upper canopy was not compensated by a larger canopy leaf area in late summer. Comparison of canopy *A*_*gross*_ with climatic variables shows that the beginning of the canopy *A*_*gross*_ decline occurred almost six weeks sooner than the seasonal declines in air temperature or incident radiation (**Figure S4**).

### Leaf carbohydrates had strong diurnal dynamics, while rhizome carbohydrate followed strong seasonal dynamics

**Figure 5:**
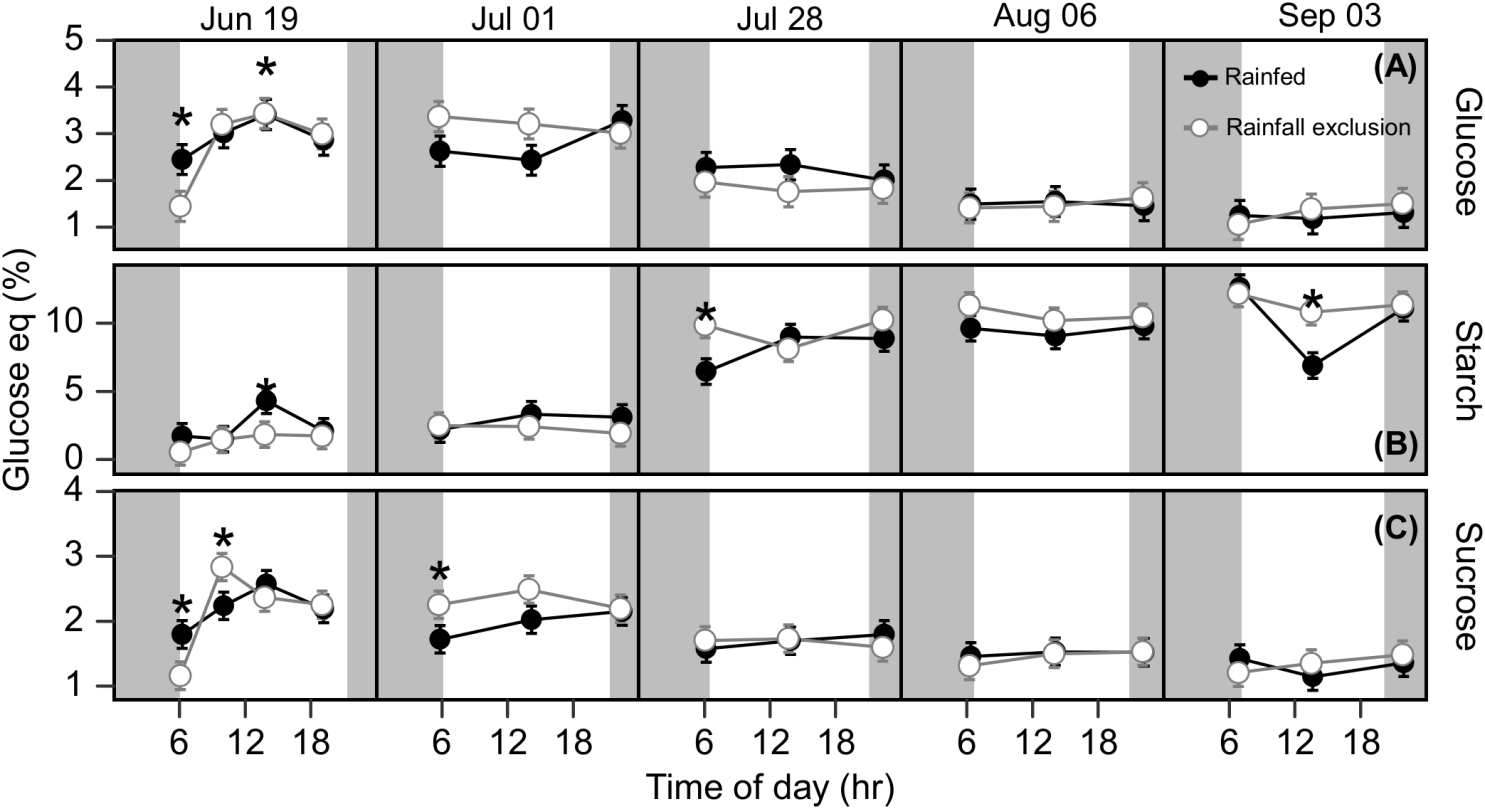
Rhizome free glucose **(A)**, starch **(B)** and sucrose **(C)** at each timepoint during the day, over the course of the growing season, for plants grown inside (white fill, grey line) and outside (black fill, black line) rainfall exclusion shelters. Asterisks indicate a significant difference between treatments (P< 0.05). Data are mean ± S.E. (n = 4).

Under ambient rainfall, leaf carbohydrates had no clear seasonal dynamics but did show clear diurnal dynamics (**Figure S5**). Leaf glucose and sucrose followed the diurnal pattern of photosynthesis, being lower in morning and afternoon samplings, and higher at noon. Leaf starch, in contrast, tended to accumulate during the day; its accumulation rate was ~7-fold higher than the other carbohydrates when expressed on a glucose-equivalent basis (**Figure S5**; **Table S1**). September 4 showed the largest fluctuation in rhizome starch in the ambient treatment. It is unclear if this midday decrease in rhizome starch is of physiological meaning or a product of sample variability. Overall, rhizome carbohydrate concentrations remained constant during the day and changed over the course of the season (**Figure 5**). Glucose and sucrose concentrations decreased from late July onward, while the starch concentration showed a corresponding increase (**Figure 5** and **Table 1S**). Rhizome glucose decreased from 3.1% in early summer to 1.5% in late summer (52% decrease; P< 0.0001; **Figure 5**), while rhizome sucrose decreased from 2.2% to 1.6% (28% decrease; P = 0.022; **Figure 5**). Starch increased from 2.6% in early summer to 9.9% in late summer (3.94-fold increase; P< 0.0001; **Figure 5**).

### End-of-season biomass was 33% lower, and soil water content was ~27% lower under the rainfall exclusion shelters

The 2020 growing season witnessed several large rain events (**Table 1**; **Figure S6A**). Rainfall exclusion shelters successfully blocked rainfall and imposed water limitation for the switchgrass plots. Soil VWC was consistently lower under the rainfall exclusion shelters at the 10-cm and 25-cm depths (**Table 1**; **Figure S6B**). After each rain event, soil VWC under the shelters was at least 2-fold, and on average 3.7-fold, lower than outside the rainfall exclusion shelters. End-of-season aboveground biomass of the switchgrass was 4.67 ± 0.52 kg m^-2^ outside the rainfall exclusion shelters and 2.95 ± 0.52 kg m^-2^ under the rainfall exclusion shelters (~37% decrease; P = 0.059).

**Table 1:** Total monthly precipitation (mm) and soil volumetric water content (VWC; m3/m3) in 2020 at the rainfall exclusion shelters Biofuel Cropping System Experiment (42.394290, -85.374126) and historic record (2009-2018) between shelter deployment (May 27th) and last sampling date (Sept 3rd).

		Precipitation (mm)			soil VWC (m^3^/m^3^)	
Month	Days	2020	2009-2018		2020	2009-2018
May	5	8	20		0.35	0.33
Jun	30	86	78		0.25	0.33
Jul	31	50	82		0.22	0.3
Aug	31	114	86		0.25	0.31
Sep	5	30	9		0.28	0.32
Total		288	275	Average	0.27	0.32

The 2020 data were collected from Soil VWC measured at 0.25 m depth in 2020 and at 0.30 m depth for 2009-2018 period. Total precipitation and average soil VWC for the entire period of study is also presented.

### Imposed water-deficit conditions decreased leaf photosynthesis, *ϕPSII* , and leaf water potential

The imposed season-long water deficit reduced leaf photosynthesis during the growing season. Plants under the shelters showed 12% and 26% lower $A_{net}^{'}$ on July 1 (P = 0.059) and September 3, respectively, compared to outside of the shelters (P = 0.030; **Figure 2**). These differences were mainly driven by reductions in *A*_*net*_ at noon, which decreased by 14% and 38% on July 1 (P = 0.023) and September 3, respectively (P< 0.0001; **Figure 2**). In addition, *A*_*net*_ under the shelters was 22% lower in the mid-morning on September 3 (P = 0.023). Similarly, plants under the shelters had reduced *ϕPSII* on the same dates and timepoints of the day; *ϕPSII* was on average ~5% lower under the shelters on July 1 (P = 0.013) and September 3 (P< 0.0049; **Figure 3**).

**Figure 6:**
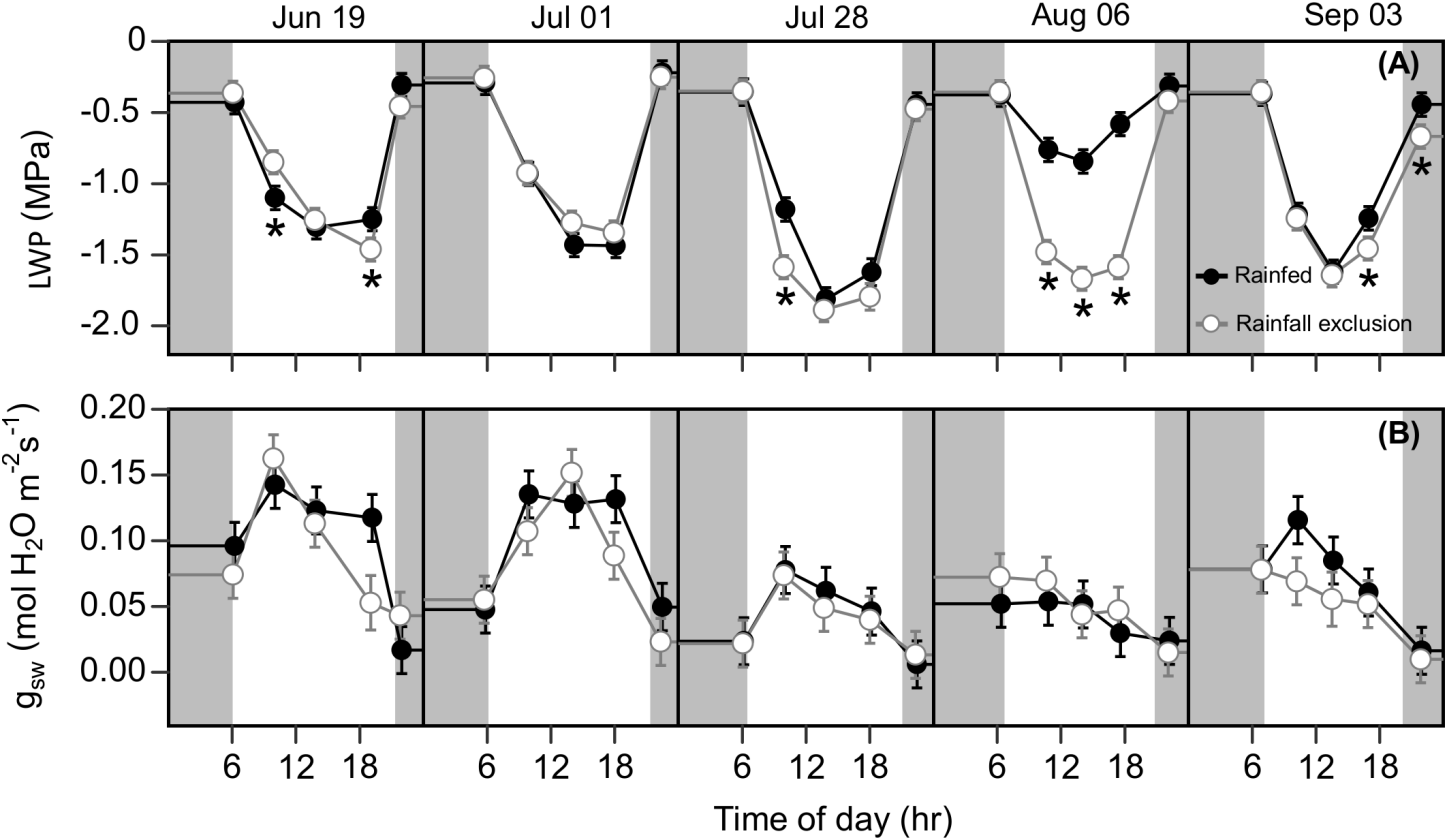
Switchgrass leaf water potential ( *LWP* ; **(A)** and stomatal conductance to water ( *g*_*sw*_ ; **(B)** at each timepoint during the day, over the course of the growing season, for plants grown under (white fill, grey line) and outside (black fill, black line) rainfall exclusion shelters. Asterisks indicate a significant difference between treatments (P< 0.05). Data are mean ± S.E. (n = 4).

Water-deficit effects on LWP were consistent over the season, and we found significant effects for at least one sampling timepoint in 4 out of the 5 sampling dates (**Figure 6A**). Plants under the rainfall exclusion shelters had 12 – 37% lower LWP than rainfed plants. These differences only corresponded with significant effects on *A*_*net*_ and $A_{net}^{'}$ on September 3 after a large precipitation event. No water-deficit effects were found on stomatal conductance ( *g*_*sw*_) during the day or across the growing season (P > 0.1; **Figure 6B**).

### Imposed water-deficit conditions had only marginal effects on leaf glucose accumulation, and no effects on rhizome carbohydrates

Rhizome carbohydrates were not affected significantly by the imposed water-deficit conditions in most comparisons (**Figure 5**). Leaves had on average 12 – 22% higher mean sucrose and starch concentrations outside the shelters, but the differences were not significant (P_glucose_ = 0.13; P_sucrose_ = 0.17; P_starch_ = 0.015; **Figure S5**). In late summer, plants outside the shelters had higher leaf glucose and starch accumulation rates than under the shelters (**Table S1**). Sucrose accumulation rates in the leaf were positive in plants outside the shelters until July 28 and then became almost null until the end of the experiment (**Table S1**). In turn, sucrose accumulation in plants under the shelters followed the opposite pattern, i.e., positive only after July 28 (**Table S1**).

## Discussion

Switchgrass carbon source-sink balances markedly changed during the growing season. In early summer under ambient rainfall, source indicators (i.e., leaf photosynthesis: *A*_*net*_ , $A_{net}^{'}$ , *ϕPSII* , ETR; **Figures 1**, **2**, **4**) were at their seasonal maximum, and sink indicators (i.e., leaf and rhizome carbohydrate concentrations) were at their seasonal minimum (**Figures 1**, **5**). As the season progressed, switchgrass plants transitioned to lower photosynthetic activity ( *A*_*net*_ and $A_{net}^{'}$ decreased by ~ 50%) and higher sink activities (4-fold increase in rhizome starch; **Figure 5**). This seasonal decline in photosynthetic rates was also observed at the canopy level based on eddy covariance estimates of canopy *A*_*gross*_ (**Figure 4**) at a companion site. Exclusion of rainfall by shelters across the entire growing season showed that water limitation did not alter these dynamics; the photosynthetic decline and carbohydrate accumulation showed similar onsets and magnitudes to the rainfed plants (**Figures 2**, **3**, **5**; **Figure S4**). While late-season rainfalls increased switchgrass photosynthesis, they did not restore $A_{net}^{'}$ to early-summer values, which supports the hypothesis that decreasing rhizome sink demand drives the seasonal photosynthetic decrease more than does decreasing water availability.

### Sink limitations

The late summer photosynthetic decline may be driven by sink limitations triggered by rhizome carbohydrate buildup. As perennial grass biomass allocation to flowers and seeds is minimal (Boe, 2007; Giannoulis et al., 2016), mature stands may lack major sinks for photosynthates once vegetative growth has ceased (Tejera et al., 2021; Tejera et al., 2022) and rhizome carbohydrate accumulation is complete. Rhizome carbohydrate concentrations and leaf photosynthesis were inversely correlated, and as rhizome starch reached maximum accumulation in mid-summer, leaf photosynthesis declined to low rates (**Figures 1**, **5**). Similar seasonal patterns have been reported in other perennial grasses. In sugarcane (*Saccharum* sp.), sucrose (the carbohydrate storage molecule for that species) increased 5-fold in the main sink organ during summer while *A*_*net*_ decreased by >50% (De Souza et al., 2018), and in *Miscanthus × giganteus* starch content in the leaf increased by 8-fold by the end of the growing season while *A*_*net*_ decreased ~50% (De Souza et al., 2013). The findings of our study on switchgrass support the sink limitation hypothesis and resolve the interacting and potentially conflicting effects of source-sink relations and water deficit on seasonal changes of photosynthesis. Our results showed that water deficit conditions later in the season had insignificant effects on the photosynthetic decline.

The decrease in leaf photosynthesis was accompanied by a decrease in *ϕPSII*  (**Figures 1**, **3**), suggesting that autumnal senescence was ongoing. Autumnal senescence is characterized by a decrease in chlorophyll content and an up-regulation of genes associated with protein degradation (Palmer et al., 2015; Palmer et al., 2019). During this process, light-harvesting pigments of the photosynthetic system are degraded (Moy et al., 2015), leading to N retranslocation from aboveground to belowground organs (Yang et al., 2009; Yang et al., 2016; Yang and Udvardi, 2018; Massey et al., 2020), and a concomitant decrease in photosynthetic capacity (Tang et al., 2005; Galvagno et al., 2013; Donnelly et al., 2020). Therefore, the decline in leaf photosynthesis may not be driven solely by sink limitations but also a decrease in photochemical efficiency of photosynthesis resulting from senescence. Note that sink limitations and senescence are not mutually exclusive mechanisms but rather could be a coherent response triggered by the environment or carbohydrate buildup and sink limitations. Sugar and/or starch accumulation is known to trigger leaf senescence in maize, trees, and other species, but this has not been tested in perennial grasses (Noodén et al., 1997; Wingler and Roitsch, 2008; Holland et al., 2016).

### Water-deficit

Experimentally imposed water stress affected switchgrass photosynthesis both in early and late summer. If water availability had been the sole driver of the late-season photosynthesis decline, switchgrass plants outside the shelters, where soil water was replenished after precipitation events, would have shown different seasonal dynamics (e.g., later onset of the decline or no decline). In our experiment, we found that late-season rainfall increased switchgrass photosynthesis, but the magnitude of the effect was not enough to compensate for the seasonal decline. In comparison, the imposed water limitation had marginal effects on rhizome carbohydrate accumulation; concentrations from plants under and outside the rainfall exclusion shelters were almost identical (**Figure 5**). Leaf carbohydrates were less resilient to water deficit; during the growing season switchgrass leaves under the shelters had 12 – 22% lower sucrose and starch content (**Figure S5**). Carbohydrate accumulation rate, as a direct proxy for sink activity, was also higher under the shelters than outside the shelter in early-summer (**Table S1**). These differences in leaf starch corresponded with some differences in $A_{net}^{'}$ . Our interpretation is that plants under the shelter with lower *A*_*net*_ had less carbon available for sucrose and starch synthesis, leading to lower sucrose and starch in the leaf (**Figure S5**). With lower carbohydrate concentrations in the leaf, switchgrass growing under the shelters would need to adjust carbohydrate mobilization and degradation through the night to avoid carbon starvation at the end of the night (Smith and Stitt, 2007). This reduction in carbohydrate mobilization and degradation at night may drive the lower nocturnal respiration rates observed on July 1 and August 6.

Water deficit effects on switchgrass are mainly studied using leaf photosynthesis and physiological responses, while effects on sink activities are not as well understood. Few studies reporting changes in leaf carbohydrate under drought conditions in switchgrass suggest that the response may be specific to the particular storage carbohydrate. Key carbohydrates such as trehalose, fructose (Liu et al., 2015), and proline (Kim et al., 2016; Hoover et al., 2018) seem to be more readily affected, while other soluble sugars such as glucose or sucrose as well as starch remain relatively constant (Hoover et al., 2018). Our work is distinct from earlier water deficit studies because we present diurnal and seasonal leaf carbohydrate measurements. We found that the small effects of water deficit on diurnal dynamics of leaf carbohydrates did not show a clear relationship with the seasonal course. Our results suggest that starch, in addition to accumulating in the rhizomes during the growing season, acts as a transitory carbohydrate storage molecule in switchgrass leaves, accumulating during the day and presumably being consumed overnight.

### Switchgrass resilience to drought

Switchgrass photosynthesis was resistant to water limitation. Even when soil VWC dropped by ~4-fold under the shelters, $A_{net}^{'}$ and *A*_*net*_ decreased by up to ~26%. Other studies have found a stronger negative leaf photosynthesis response to water deficit (Barney et al., 2009; Lovell et al., 2016; Taylor et al., 2016; Hawkes and Kiniry, 2018; Chen et al., 2020), including across 49 switchgrass genotypes in which $A_{\mathrm{net}}$ decreased by 40 – 80% under drought stress (Liu et al., 2015). This discrepancy may be because the control treatment in our field experiment (i.e., plants outside the shelter) experienced ambient environmental conditions, while other studies used irrigated plants as the control. While the latter is useful to characterize the response, our approach is more consistent with the natural environment and allows a better characterization of the seasonal dynamics.

In light of our results, it may seem counterintuitive that, at the whole-plant level, end-of-season aboveground biomass was reduced by 33% by the shelters. The switchgrass strategy to cope with water deficit stress may rely on whole-plant responses, such as modifying the number of tillers and/or leaves per tiller, producing smaller and thinner stands under the shelter while maintaining leaves that had similar photosynthetic performance to leaves outside the shelters. Alternatively, differences in end-of-season biomass could be driven by switchgrass stands with similar tiller density and leaf area, but small (and often insignificant) differences in leaf photosynthesis accumulate over the growing season yielding larger differences in end-of-season biomass.

### The presence of late-season decline in photosynthesis as an improvement strategy for switchgrass and other perennials

The switchgrass photosynthetic decline over the growing season, which we observed in both plot and field-scale switchgrass stands, reveals a potential for yield improvement. Using simple linear interpolation between sampling dates, the upper canopy of switchgrass assimilated ~45 mol CO_2_ m^-2^ during the growing season. If leaf photosynthesis had remained constant at early summer levels during the 6 weeks when the environment was still favorable, switchgrass would have assimilated an additional 52% carbon. Scaling leaf-level photosynthesis to end-of-season biomass is beyond the scope of this study, but assuming end-of-season biomass is proportional to the accumulated CO_2_ during the study period, this forgone 52% of CO_2_ assimilation could potentially lead to an extra ~1.2 Mg ha^-1^ of end-of-season aboveground biomass. While these calculations oversimplify the relationship between gross carbon fixation and biomass production, the physiological understanding of this decline could elucidate ways to maintain higher photosynthetic rates for longer in the season and thereby increase biomass yields. This late-season decline in photosynthesis is commonly observed in many perennial grasses, and therefore improvement strategies could have a broader impact on cropping systems that include perennial grasses and possibly trees (Kosugi and Matsuo, 2006; Boersma et al., 2015; De Souza et al., 2018; Kar et al., 2020; Stavridou et al., 2020).

## Conclusions

Switchgrass leaf photosynthesis decreased by ~50% during the latter half of the growing season. This seasonal photosynthetic decline appears to be common across several perennial grasses, but the underlying mechanisms and the potential implications for management are not well understood. Our results suggest that this decline is not entirely driven by water limitation since leaf photosynthesis outside the rainfall exclusion shelters remained at low values even after heavy rain events in late-summer and early spring. Eddy covariance data shows that the decline also occurred at the whole-field scale and represents a physiological response that occurs even when environmental conditions remain favorable for photosynthesis. We suggest sink limitation as an important driving mechanism of the photosynthetic decline, as rhizome starch reached peak concentrations around the same time that leaf photosynthesis fell to lower rates. If sink limitation were the leading cause of the seasonal photosynthesis decline, then strategies to alleviate sink limitation could be included in switchgrass breeding programs with the goal of increasing yields.

## Data availability statement

The raw data supporting the conclusions of this article will be made available by the authors, without undue reservation.

## Author contributions

All authors contributed ideas; MT-N collected and analyzed field data and led the writing of the manuscript. MA and JC maintained eddy covariance towers, and collected and curated the data. SH and GR contributed to conception and design of the experiment. BW oversaw and provided substantial contributions to the project. All authors contributed to the article and approved the submitted version.
