# Corrigendum: Seasonal decline in leaf photosynthesis in perennial switchgrass explained by sink limitations and water deficit

**DOI:** 10.3389/fpls.2023.1204150

**Published:** 2023-04-20

**Authors:** Mauricio Tejera-Nieves, Michael Abraha, Jiquan Chen, Stephen K. Hamilton, G. Philip Robertson, Berkley James Walker

**Affiliations:** ^1^ MSU-DOE Plant Research Laboratory, Michigan State University, East Lansing, MI, United States; ^2^ Great Lakes Bioenergy Research Center, Michigan State University, East Lansing, MI, United States; ^3^ W. K. Kellogg Biological Station, Michigan State University, Hickory Corners, MI, United States; ^4^ Center for Global Change and Earth Observations, Michigan State University, East Lansing, MI, United States; ^5^ Department of Geography, Environment, and Spatial Sciences, Michigan State University, East Lansing, MI, United States; ^6^ Department of Integrative Biology, Michigan State University, East Lansing, MI, United States; ^7^ Department of Plant, Soil, and Microbial Sciences, Michigan State University, East Lansing, MI, United States; ^8^ Department of Plant Biology, Michigan State University, East Lansing, MI, United States

**Keywords:** photosyhthesis, storage carbohydrates, sink limitation, perennial grass, drought, circadian, source- sink- relationships, C4 photosynthesis

In the published article, an author name was incorrectly written as Berkley Walker James. The correct spelling is Berkley James Walker.

The authors apologize for this error and state that this does not change the scientific conclusions of the article in any way. The original article has been updated.

