# Supplementary material for: Seasonal decline in leaf photosynthesis in perennial switchgrass explained by sink limitations and water deficit
Source: Front Plant Sci. 2023 Jan 4;13:1023571. doi: 10.3389/fpls.2022.1023571 (PMC9846045; doi:10.3389/fpls.2022.1023571)
Supplement: Supplementary file 1 [file DataSheet_1.docx]

Supplementary Material

Seasonal decline in leaf photosynthesis in perennial switchgrass explained by sink limitations

Mauricio Tejera-Nieves^1,2^, Michael Abraha^2, 3, 4^, Jiquan Chen^2, 4, 5^, Stephen K. Hamilton^2,3,6^, G. Philip Robertson^2,3,7^, Berkley Walker^1,2,8*^

^1^ MSU-DOE Plant Research Laboratory, Michigan State University, East Lansing, MI, USA

^2^ Great Lakes Bioenergy Research Center, Michigan State University, East Lansing, MI, USA

^3^ W. K. Kellogg Biological Station, Michigan State University, Hickory Corners, MI, USA

^4^ Center for Global Change and Earth Observations, Michigan State University, East Lansing, MI, USA

^5^ Department of Geography, Environment, and Spatial Sciences, Michigan State University, East Lansing, MI, USA

^6^ Department of Integrative Biology, Michigan State University, East Lansing, MI, USA

^7^ Department of Plant, Soil, and Microbial Sciences, Michigan State University, East Lansing, MI, USA

^8^ Department of Plant Biology, Michigan State University, East Lansing, MI, USA

^*^Corresponding author: [berkley@msu.edu](mailto:berkley@msu.edu)

# Supplementary Figures and Tables

## Supplementary Figures


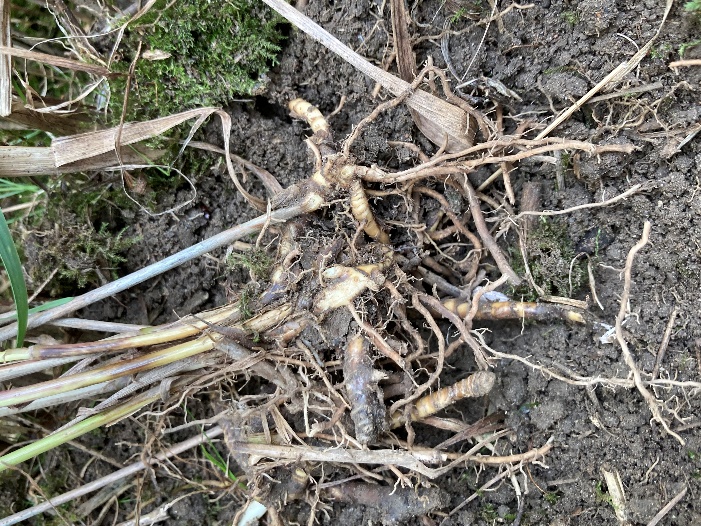


**Supplementary Figure 1.** Unearthed switchgrass belowground network of rhizomes and roots. Red circles indicate typical rhizomes used for carbohydrate sampling. Photo credit: Mauricio Tejera-Nieves


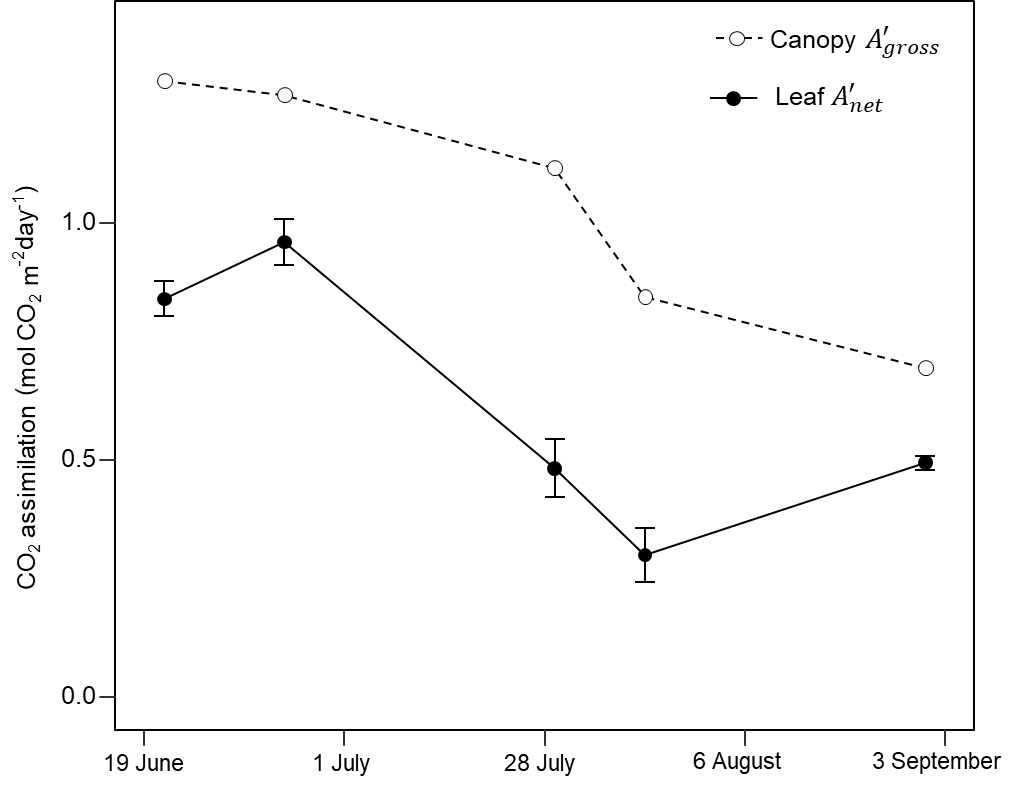


**Supplementary Figure 2.** Daily net CO_2_ accumulation at the leaf level (Leaf A_net_; solid line and filled circles) and daily gross primary production at the canopy level (Canopy A_gross_; dashed line and open circles) at the sampling dates. Gross primary assimilation was estimated from net ecosystem CO_2_ exchange (NEE) observations using eddy covariance method located 11 km from the experimental site.


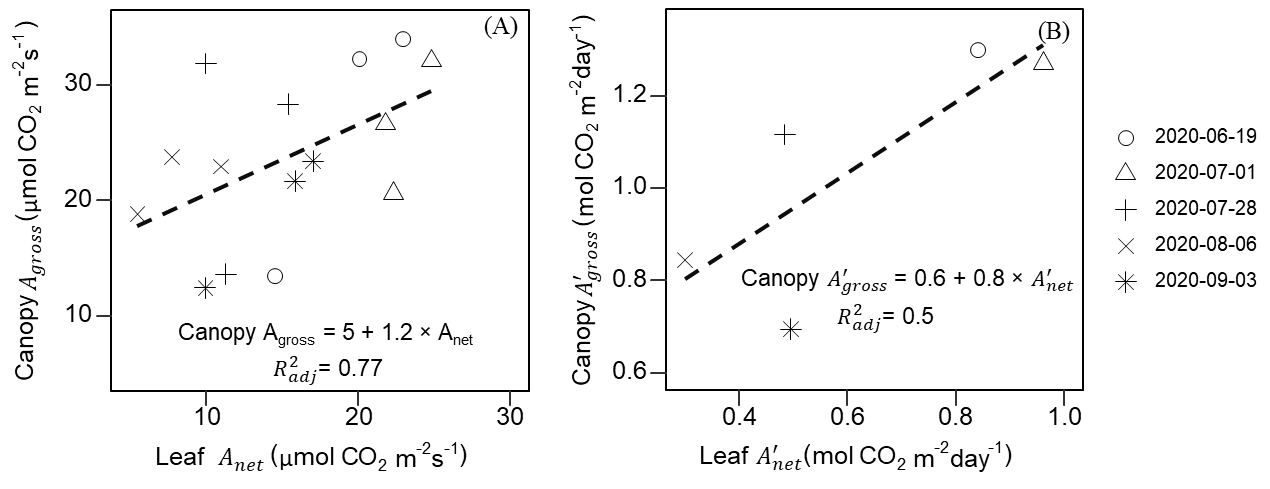


**Supplementary Figure 3:** Linear correlation between net CO_2_ assimilation at the leaf level (Leaf A_net_) and gross CO_2_ assimilation at the canopy level (Canopy A_gross_) across all measurements (A) and daily integrals (B). Equation of the linear regression (dashed line) and adjusted R^2^ are provided. Canopy A_gross_ was estimated from net ecosystem CO_2_ observations made using eddy covariance method located 11 km from the experimental site.


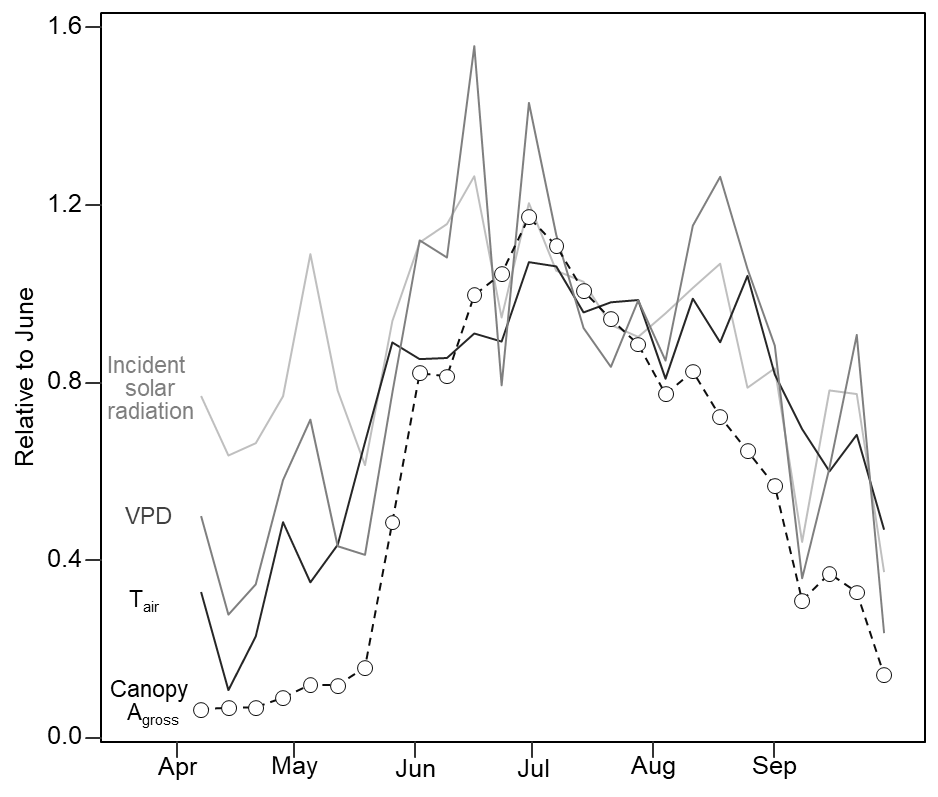


**Supplementary Figure 4:** Weekly canopy CO_2_ gross assimilation (Canopy A_gross_; dashed line and open circles), air temperature (T_air_; black), vapor pressure deficit (VPD; grey) and incident solar radiation (Solar; light grey) during the 2020 growing seasons. Values are relative to June average. Canopy A_gross_ and weather variables were measured from an Eddy Covariance tower located 11 km from the experimental site.


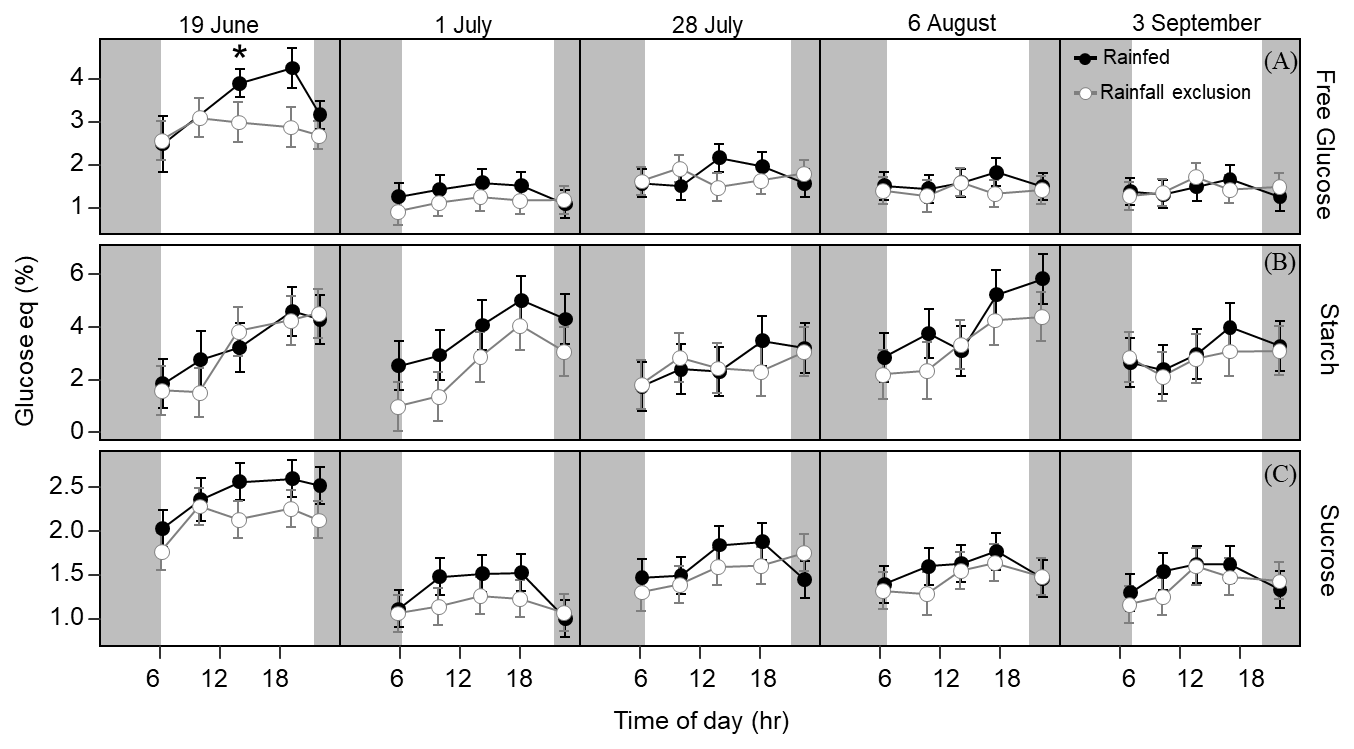


**Supplementary Figure 5:** Leaf free glucose (A), starch (B) and sucrose (C) at each timepoint during the day, over the course of the growing season, for plants grown inside (white fill, grey line) and outside (black fill, black line) rainout shelters. Asterisks indicate a significant difference between treatments (P < 0.05). Data are mean $\pm$ S.E. (n = 4; except for free glucose on 19 June, n = 2 or 3).


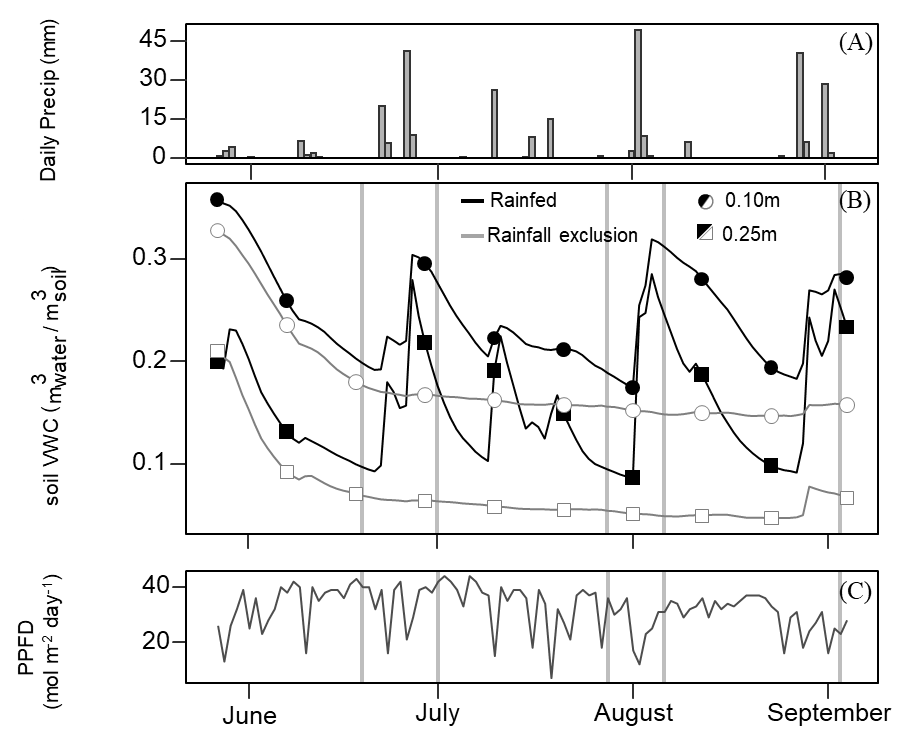


**Supplementary Figure 6:** Daily precipitation (A); daily averaged soil volumetric water content (soil VWC; B) inside (grey line, open symbols) and outside (black line, filled symbols) the rainfall exclusion shelters at 0.10 m (square) and 0.25m (circle) depths; and daily incident photosynthetic photon flux density (PPFD; C) in Biofuel Cropping System Experiment (BCSE), located in Hickory Corners, Michigan, USA (42.394290, -85.374126). Vertical grey lines indicate sampling dates.

## Supplementary Tables

| Supplementary Table 1: Switchgrass leaf and rhizome glucose, starch and sucrose accumulation rate (g kg^-1^ hour ^-1^) for rainfed plants and plants grown inside rainout shelters. Different letters indicate significant differences between treatments (p-value < 0.05) and asterisks indicate rates significantly different from zero (p-value < 0.05). Data are mean ± S.E. (n = 4). | | | | | | |
| --- | --- | --- | --- | --- | --- | --- |
|  | Glucose accumulation rate | | Starch accumulation rate | | Sucrose accumulation rate | |
| Date | Rainfed | Rainfall exclusion | Rainfed | Rainfall exclusion | Rainfed | Rainfall exclusion |
| Leaf | | | | | | |
| 19 Jun | 1.20 ± 0.37 a* | 0.25 ± 0.27 b | 2.1 ± 0.54 * | 2.40 ± 0.53 * | 0.40 ± 0.14 * | 0.29 ± 0.14 * |
| 1 Jul | 0.20 ± 0.18 | 0.24 ± 0.18 | 2.0± 0.57 * | 2.60 ± 0.57 * | 0.29 ± 0.14 * | 0.16 ± 0.14 |
| 28 Jul | 0.43 ± 0.19 a* | -0.11 ± 0.19 b | 1.4 ± 0.58 * | 0.31 ± 0.59 | 0.38 ± 0.14 * | 0.27 ± 0.14 |
| 6 Aug | 0.24 ± 0.20 | 0.048 ± 0.20 | 1.8 ± 0.63 * | 1.80 ± 0.63 * | 0.30 ± 0.15 | 0.36 ± 0.16 * |
| 3 Sep | 0.30 ± 0.23 | 0.28 ± 0.23 | 1.4 ± 0.70 | 0.24 ± 0.70 | 0.29 ± 0.17 | 0.40 ± 0.17 * |
| Rhizome | | | | | | |
| 19 Jun | 0.43 ± 0.52 | 0.98 ± 0.52 | 0.76 ± 1.3 | 0.86 ± 1.3 | 0.36 ± 0.34 | 0.55 ± 0.34 |
| 1 Jul | -0.23 ± 0.84 | -0.21 ± 0.85 | 1.40 ± 2.0 | -0.12 ± 2.1 | 0.32 ± 0.55 | 0.31 ± 0.56 |
| 28 Jul | 0.061 ± 0.91 | -0.30 ± 0.92 | 3.20 ± 2.2 | -2.50 ± 2.2 | 0.14 ± 0.60 | 0.051 ± 0.61 |
| 6 Aug | 0.11 ± 0.90 | -0.0049 ± 0.90 | -0.20 ± 2.2 | -1.70 ± 2.2 | 0.11 ± 0.60 | 0.21 ± 0.60 |
| 3 Sep | -0.16 ± 1.00 | 0.50 ± 1.10 | -8.00 ± 2.6 | -2.30 ± 2.6 | -0.45 ± 0.70 | 0.20 ± 0.70 |
